# Repositioning of bromocriptine for treatment of acute myeloid leukemia

**DOI:** 10.1186/s12967-016-1007-5

**Published:** 2016-09-07

**Authors:** María Carmen Lara-Castillo, Josep Maria Cornet-Masana, Amaia Etxabe, Antònia Banús-Mulet, Miguel Ángel Torrente, Meritxell Nomdedeu, Marina Díaz-Beyá, Jordi Esteve, Ruth M. Risueño

**Affiliations:** 1 Centre Esther Koplowitz, Josep Carreras Leukaemia Research Institute, Campus Clínic-University of Barcelona, Rosselló 149-153, 08036 Barcelona, Spain; 2 Department of Hematology, Hospital Clínic, Institut d’Investigacions Biomèdiques August Pi i Sunyer (IDIBAPS), Barcelona, Spain; 3 University of Barcelona, Barcelona, Spain

**Keywords:** AML, Bromocriptine, New drug, Drug repositioning, Leukemia stem cells

## Abstract

**Background:**

Treatment for acute myeloid leukemia (AML) has not significantly changed in the last decades and new therapeutic approaches are needed to achieve prolonged survival rates. Leukemia stem cells (LSC) are responsible for the initiation and maintenance of AML due to their stem-cell properties. Differentiation therapies aim to abrogate the self-renewal capacity and diminish blast lifespan.

**Methods:**

An in silico screening was designed to search for FDA-approved small molecules that potentially induce differentiation of AML cells. Bromocriptine was identified and validated in an in vitro screening. Bromocriptine is an approved drug originally indicated for Parkinson’s disease, acromegaly, hyperprolactinemia and galactorrhoea, and recently repositioned for diabetes mellitus.

**Results:**

Treatment with bromocriptine reduced cell viability of AML cells by activation of the apoptosis program and induction of myeloid differentiation. Moreover, the LSC-enriched primitive AML cell fraction was more sensitive to the presence of bromocriptine. In fact, bromocriptine decreased the clonogenic capacity of AML cells. Interestingly, a negligible effect is observed in healthy blood cells and hematopoietic stem/progenitor cells.

**Conclusions:**

Our results support the use of bromocriptine as an anti-AML drug in a repositioning setting and the further clinical validation of this preclinical study.

**Electronic supplementary material:**

The online version of this article (doi:10.1186/s12967-016-1007-5) contains supplementary material, which is available to authorized users.

## Background

Acute myeloid leukemia (AML) is the most common acute leukemia in adults and the survival rate 5 years after diagnosis is below 50 % according to data collected from SEER Epidemiologic Datasets [1]. Standard treatment consists of the combination of an anthracycline and cytarabine but, despite great improvement in patient management and hematopoietic cell transplantation, treatment regimens have been substantially unchanged for the last decades [1]. Indeed, current available chemotherapy may have reached its limits. In this context, drug repositioning appears as a promising strategy for AML as safety and pharmacokinetic profiles of candidates are well-known, enabling a faster bench-to-bedside transition [2].

Leukemia stem cells (LSC) have been described in the majority of AML patients and constitute a cell fraction with self-renewal and differentiation properties [3]. Due to their stem-cell like properties, relapse episodes and refractoriness to treatment were associated to LSC function [4]. Therefore, eradication of AML requires the elimination of the LSC population. As the equilibrium between self-renewal and differentiation is tightly regulated, induction of terminal differentiation of LSCs will inhibit self-renewal capacity and eventually contributing to AML cell population consumption [5]. Additionally, more differentiated AML blasts are more sensitive to currently used chemotherapeutics [6]. Thus, differentiation therapies hold promise as a therapeutic approach to target LSCs.

By means of an in silico screening searching for approved small bioactive drugs that potentially induce differentiation of AML cells, bromocriptine, a drug originally indicated for Parkinson’s disease, acromegaly, hyperprolactinemia and galactorrhoea, and recently repositioned for diabetes mellitus [7, 8], was identified. Although bromocriptine was originally described as a dopamine receptor agonist/antagonist [9], its mechanism of action for each disease is partially unknown. Here, we demonstrated that bromocriptine has a differential anti-leukemia activity, sparing healthy blood cells.

## Methods

### In silico screening

Gene signature associated with PMA (phorbol myristate acetate)-induced differentiation in HL60 cells was obtained from GSE982 and analysed as described previously (vehicle-control treated vs. PMA-treated samples) [10, 11]. Briefly, raw files (.cel) were normalized using GenePattern software (Broad Institute Cancer Program; http://www.broadinstitute.org/cancer/software/genepattern/) and probe set with a differential expression of at least twofold of change and *p* value below 0.005 were chosen. The 481-top-ranking upregulated (Additional file 1: Table S1) and 500-top-downregulated (Additional file 2: Table S2) probes during PMA treatment were selected for in silico signature-based screening (Connectivity Maps; http://www.broadinstitute.org/cmap/) [12]. The results obtained were filtered at a *P* value <0.05 and a connectivity score >0.75 in HL-60 (AML cell line) but <0.5 in PC3 (prostate cancer cell line) and MCF7 (breast cancer cell line), at a concentration <10 µM.

### AML cell lines

AML cell lines HL60 (ACC-3) [13], KG-1 (ACC-14) [14], MonoMac-1 (ACC-252) [15] and Kasumi-1 (ACC-220) [16] were obtained from DSMZ (Deutsche Sammlung von Mikroorganismen und Zellkulturen) and the human stroma cell line HS-5 was purchased from ATCC (American Type Culture Collection). Experiments were performed within 6 months after receipt or recovery after thawing. AML cell lines were cultured in complete RPMI medium (PAA laboratories) supplemented with 10 % fetal bovine serum (Lonza), sodium pyruvate (Lonza) and non-essential amino acids (Lonza) according to manufacturers’ recommendations. HS-5 cell line was cultured in complete DMEM medium (PAA laboratories) supplemented with 10 % fetal bovine serum (Lonza) prior to co-culture experiments. Co-culture experiments were performed in complete RPMI medium as described for AML cells.

### Primary samples

Peripheral blood (PB) and bone marrow (BM) primary AML samples were obtained from patients diagnosed with AML at Hospital Clínic of Barcelona (Spain) (Table 1). AML diagnosis and classification was based on accepted WHO criteria [17]. All patients provided written informed consent in accordance with the Declaration of Helsinki, and the study was approved by the Ethics Committee of Hospital Clínic of Barcelona. Blood mature mononuclear cells (MNCs) were isolated from healthy-donor buffy coats provided by Banc de Sang i Teixits (Barcelona, Spain). Umbilical cord blood MNCs were obtained after Ficoll (GE) density gradient centrifugation and were depleted for lineage marker-positive cells (Miltenyi). Primary AML blasts were cultured in IMDM (PAA laboratories) supplemented with 3 % heat-inactivated fetal bovine serum (Lonza), 1 × BIT (StemCell Technologies), 5 ng/ml IL3 (Peprotech), sodium pyruvate (Lonza) and β-mercaptoethanol (Sigma).

**Table 1:** AML patients’ information

AML sample	Gender	Age (year)	WHO subtype	WBC count (× 10^9^/L)	% Blasts in PB	% Blasts in BM	Karyotype	Additional molecular features	ELN Risk group	FAB
#2	M	28	AML without maturation	143	98	90	N/A	FLT3-ITD	Intermediate	M1
#3	M	40	AML without maturation (AML with mutated CEBPA–provisional entity-)	52	66	80	46,XY	Biallelic CEBPA mutation	Favorable	M1
#4	F	34	AML with myelodysplasia-related changes	32	16	44	45,XX, -7	FLT3wt and NPMwt	Adverse	M4
#5	M	45	AML with t(6,9)(p23;q34); DEK-NUP214	40	58	43	46,XY,t(6;9)(p23;q34)	FLT3-ITD	Adverse	M0
#6	M	48	AML with t(8;21)(q22;q22); *RUNX1*-*RUNX1T1*	54	5	24	46,XY,t(8;21)(q22;q22)	FLT3N/A and NPMwt	Favorable	M2
#8	M	61	AML with t(8;21)(q22;q22); *RUNX1*-*RUNX1T1*	21.4	51	89	45,X–Y,t(8;21)(q22;q22) [19] /46,XY [1]	FLT3wt and NPMwt	Favorable	M2
#9	F	58	AML with myelodysplasia-related changes	100.7	45	80	46,XX, del(5)(q23q33), t(8;9)(p11;q34) [20]	FLT3wt and NPMwt	Adverse	M5
#10	M	24	AML with myelodysplasia-related changes	7.1	83	30	46,XY [20]	FLT3wt and NPMwt	Intermediate	M1
#11	M	49	AML with myelodysplasia-related changes	76.4	42	26	46-47,XY, del(5)(q22q34), del(6)(q22q25), del(7)(q22q23),-8,-9, add(11)(q23), +i(11)(q11),-16, +mar1, +mar2, +mar3[cp8].	FLT3wt and NPMwt	Adverse	M1
#14	M	22	AML with t(8;21)(q22;q22); *RUNX1*-*RUNX1T1*	20.4	83	69	45,X,-Y,t(8;21)(q22;q22) [17]/46,XY [3]	FLT3 ITD	Favorable	M2
#22	F	60	AML with myelodysplasia-related changes	218.1	68	36	48,XX, +8, +21 [13]	FLT3wt and NPMwt	Intermediate	M4

### Analysis of cell viability

For AML cell lines, 100,000 cells were treated in 96-well plates in complete medium. Bromocriptine (Sigma) was added at the concentration indicated. Co-culture experiments were performed seeding 1:3 HS-5/AML cells. Cell viability was measured by 7-AAD (eBioscience) exclusion and positivity for Hoechst33342 (Sigma) by flow cytometry and cell count was obtained by volume in a FACSVerse or FACSCantoII cytometer (BD). In co-culture experiments, AML cells were discriminated based on CD45 expression.

In order to test the cytotoxic effect of bromocriptine on primary AML patient samples, between 100,000 and 150,000 cells were seeded in 200 µL of IMDM complete medium per well (96-well plates were used). Cells were treated once with 10 µM bromocriptine or the vehicle-control (DMSO). 24 or 72 h after treatment, cells were stained with CD45, 7-AAD, and Hoechst33342. The blast population was identified by their profile CD45 versus side scatter (SSC) [18]. Within the blast population, live cells were 7-AAD negative and Hoechs33342 positive. Number of live cells was obtained due to the volumetric count performed during the acquisition.

All experiments were performed in triplicate. Data was normalized by setting the vehicle-control treated group as 100. Each patient data set is represented normalized against its own vehicle-treated control. Consequently, each experimental point was divided by the mean of the corresponding vehicle-treated control group and expressed as a percentage. Statistical analysis (Mann–Whitney test) and IC50 determination were calculated in GraphPad (Prism software). FlowJo software (TriStar) was used for flow cytometry analysis.

### Annexin V detection

AML cells were treated with bromocriptine as described above for 48 h. Cell were stained with Annexin-V following manufactures’ recommendations (BD) and analyzed using a FACSCantoII cytometer (BD). FlowJo software (TriStar) was used for flow cytometry analysis.

### Myeloid differentiation

Cells were treated as indicated for cytotoxicity assays. Forty-eight hours after treatment, cells were stained with anti-human CD11b-PE (BD) and surface expression of the antigen was analyzed by flow cytometry (FACSVerse, BD).

### Clonogenic assay

Primary AML (50 × 10^3^) or lineage-negative cord blood cells (1 × 10^3^) cells were treated for 18 h with bromocriptine and mixed with 1 mL of MethoCult H4034 Optimum (StemCell Technologies). Colonies were screened based on morphology and cellularity at day 14 by light microscopy. BFU-E (burst-forming unit-erythroid) contains >200 erythroblasts in a single or multiple clusters. CFU-GM (colony-forming unit-granulocyte/macrophage) is composed by at least 40 granulocytes and macrophages. When only granulocytes or macrophages are found, these colonies are identified as CFU-G and CFU-M, respectively. CFU-Mix or CFU-GEM is initiated by a multi-potential progenitor that produces a colony containing erythroblasts and cells of at least two other recognizable lineages. CFU-GEM tends to produce large colonies due to their primitive nature.

## Results

**Fig. 1:**
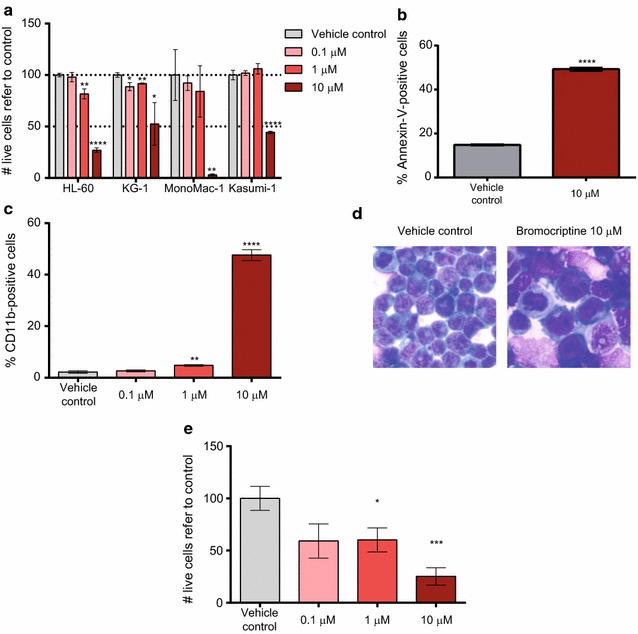
Bromocriptine treatment had an anti-leukemia activity in AML cell lines. **a** HL-60, KG-1, MonoMac-1 and Kasumi-1 AML cell lines were treated with 0.1, 1 and 10 µM bromocriptine for 48 h. Relative number of live cells refer to vehicle control-treated samples is represented. **b** HL-60 cells were treated with vehicle or 10 µM bromocriptine for 48 h. Frequency of Annexin-V-positive cells measured by flow cytometry is represented. **c** HL-60 cells were treated with bromocriptine at the concentrations indicated. Frequency of CD11b-positive cells detected by flow cytometry is represented. **d** HL-60 cells were treated with bromocriptine at 10 µM for 48 h. A representative picture of May–Grünwald–Giemsa-stained cells is shown. **e** HL-60 cells were cultured with HS-5 stroma cells at indicated concentration. Cell viability was measured 48 h after treatment by flow cytometry. *Bars* represent mean values of at least 3 experiments performed in triplicates. *Error bars* represent SEM. *p < 0.05; **p < 0.01; ***p < 0.005; ****p < 0.001

AML is characterized by the accumulation of transformed immature myeloid blasts which have lost their ability to normally differentiate. As targeted therapies aiming to force AML blast cells to terminally differentiate will ultimately result in cell death, a PMA (phorbol myristate acetate)-induced differentiation-associated gene expression profile was identified and interrogated against the Connectivity Map database (https://www.broadinstitute.org/cmap/) [12]. In order to identify potential drugs that selectively induced differentiation of AML cells, the results obtained were filtered at a *P* value <0.05 and a connectivity score >0.75 in HL-60 (AML cell line) but <0.5 in PC3 (prostate cancer cell line) and MCF7 (breast cancer cell line), at a concentration <10 µM (Additional file 3: Figure S1). Bromocriptine, a FDA- and EMA-approved drug for treatment of Parkinson’s disease [19], pituitary tumors [20], hyperprolactinemia [21] and type II diabetes [22], was identified as a potential differentiation-inducing drug for AML blasts. Four different AML cell lines (HL-60, AML FAB M2; KG-1, AML FAB M0/1; MonoMac-1, AML FAB M5 MLL-AF9 positive; Kasumi-1, AML FAB M2 t(8;21) positive) were treated at different doses of bromocriptine for 48 h. As shown in Fig. 1a, bromocriptine treatment resulted in at least 50 % cell viability reduction at 10 µM concentration. Interestingly, bromocriptine-treated AML cells were highly positive for the early apoptosis marker Annexin-V (Fig. 1b, Additional file 4: Figure S2A and data not shown), indicating that bromocriptine activated the cell death program in AML. In concordance with the in silico screening performed to identify bromocriptine, treatment with this drug induced the surface expression of the myeloid-associated differentiation marker CD11b (Fig. 1c, Additional file 4: Figure S2B and data not shown) and morphological changes compatible with terminal differentiation (Fig. 1d).

One of the principal causes of therapy failure in AML patients is the resistance to chemotherapy where the bone marrow microenvironment plays a crucial role in drug resistance and cell survival [23]. In order to investigate the effect of bone marrow (BM) stroma cells on bromocriptine-mediated cytotoxicity, AML cells were cultured in the presence of the BM stroma cell line HS-5 and treated with bromocriptine as previously described. Bromocriptine treatment overcame BM stroma-survival signaling, reducing cell viability in a dose-dependent manner (Fig. 1e). Indeed, a similar degree of cell viability reduction was induced upon bromocriptine treatment both in the presence (Fig. 1e) and absence (Fig. 1a) of HS-5 cells. Interestingly, HS-5 viability was not affected by bomocriptine treatment. Therefore, bromocriptine treatment presented an anti-leukemic cytotoxic effect accompanied by the activation of the differentiation and the apoptosis programs in a stroma-independent fashion.

**Fig. 2:**
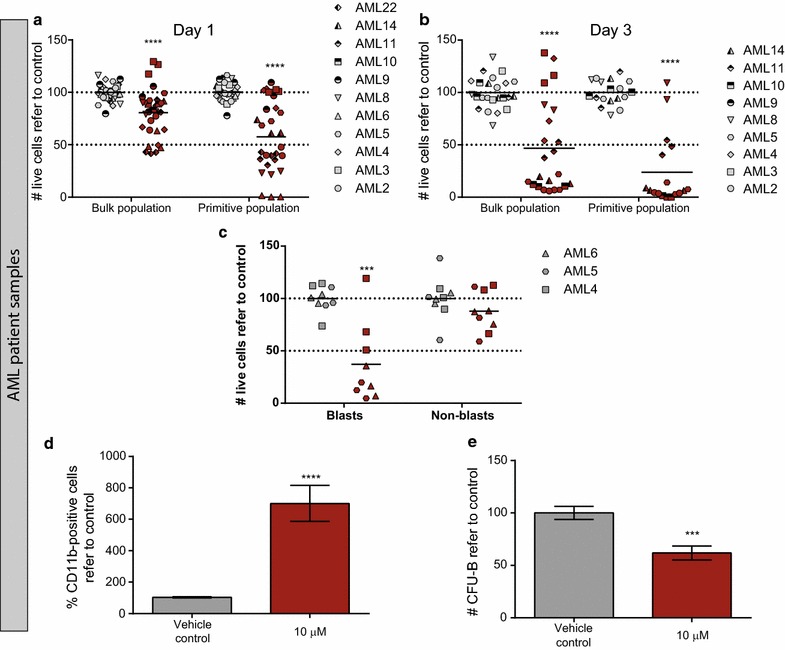
Bromocriptine treatment affected cell viability of primary AML samples, sparing healthy blood cells. AML primary patient samples were treated for 24 (**a**) or 72 (**b**) h with vehicle control or 10 µM bromocriptine. Cell viability was measured by flow cytometry. Primitive population corresponds to the CD34^+^CD38^−^ AML fraction. Live cells refer to control are represented, each *symbol* type represents an individual AML patient and each *symbol* corresponds to an independent experimental point. **c** Cell viability 72 h after 10 µM bromocriptine treatment was analyzed inside the blast and non-blast population (according to the SSC-CD45 profile). **d** Vehicle control- or bromocriptine-treated primary AML samples were assayed for the expression of CD11b. The relative frequency of CD11b-positive cells refer to control is represented. **e** Primary AML samples were treated for 18 h and cultivated in methylcellulose. 14 days after, colonies were screened based on cellularity and morphology criteria. *Bars* represent mean value of at least biological triplicates. *Error bars* correspond to SEM. CFU-B: CFU-Blasts. ***p < 0.005; ****p < 0.001

Next, the cytotoxic activity of bromocriptine was investigated in eleven primary AML patient samples, representative of several different non-APL (acute promyelocytic leukemia) subtypes. AML primary samples were treated with the vehicle control or 10 µM bromocriptine for 24 and 72 h and the cytotoxicity was measured within the blast population (identified by its side scatter profile and CD45 intensity). A significant reduction in cell viability was detected 24 h after treatment (Fig. 2a); moreover, this effect was more pronounced 72 h after treatment, when the majority of the AML blasts died upon treatment (Fig. 2b). Interestingly, treatment with bromocriptine spared non-blast cells while the blast population was severely reduced upon treatment (Fig. 2c).

AML is a hierarchical structure sustained by a “stem-cell”-like population termed LSC [3], responsible for the initiation and maintenance of the disease. Although the phenotype of this population is controversial, there is strong evidences suggesting that the CD34^+^CD38^−^ subpopulation is enriched for LSCs [4]. This primitive population displayed higher sensitivity to bromocriptine treatment than the bulk population [Fig. 2a (*p* = 0.0071) and b (*p* = 0.0081)]. Indeed, viability of this primitive cell fraction was reduced about 50 % 24 h upon treatment with bromocriptine. Next, the sensitivity to bromocriptine was analyzed according to the prognostic cytogenetic category of AML samples (Additional file 5: Figure S3) based on diagnostic karyotype [24]. No significant differences in the response to bromocriptine were observed in the bulk population among these three risk groups. Nonetheless, sensitivity to bromocriptine within the primitive population significantly decreased in the adverse risk group as compared to favorable/intermediate-risk patients, although the number of AML patients analyzed is limited.

Similarly to the effect on AML cell lines, bromocriptine induced the upregulation of CD11b, suggesting that AML blasts activated the differentiation program in the presence of the drug (Fig. 2d; Table 2).

**Table 2:** Baseline and post-treatment CD11b expression levels in primary samples from 7 AML patients

AML sample	Vehicle control treatment		Bromocriptine treatment		CD11b induction
	Mean	SEM	Mean	SEM	
#2	0.78	0.09	8.31	2.73	10.65
#4	0.82	0.49	5.00	1.90	6.10
#5	3.94	1.59	19.31	8.24	4.90
#8	3.25	0.20	14.81	11.45	4.56
#10	8.48	0.18	6.38	2.03	0.75
#14	3.37	0.14	34.02	0.95	10.09
#22	21.43	0.74	30.90	0.78	1.44

Due to the higher sensitivity to bromocriptine of the primitive cell fraction and the induction of terminal differentiation upon treatment, the clonogenic capacity as an ex vivo measure of the self-renewal and differentiation potential was interrogated in the presence of bromocriptine. Primary AML cells were treated for 18 h with bromocriptine and a CFU assay was performed. In concordance with previous results, bromocriptine also impaired the clonogenicity of AML cells (Fig. 2e).

**Fig. 3:**
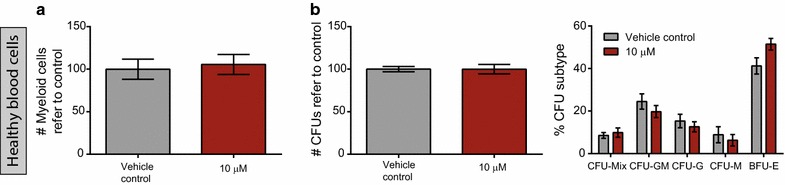
Bromocriptine treatment spared healthy blood cells. **a** Healthy CD33-positive myeloid mature cells from peripheral blood of healthy donors were treated for 72 h with vehicle control or 10 µM bromocriptine. Cell viability was measured by flow cytometry. **b** Lineage-depleted umbilical cord blood cells were treated with vehicle control or 10 µM bromocriptine for 18 h and cultured in methylcellulose. Total number of colonies (*left*) or frequency of each colony subtype (*right*) refer to control is represented. *Bars* represent mean value of at least biological triplicates. *Error bars* correspond to SEM. ***p < 0.005; ****p < 0.001

To demonstrate the differential effect of bromocriptine in AML cells versus healthy blood cells, mature myeloid cells isolated from peripheral blood of healthy donors, as AML normal counterpart, were treated with bromocriptine in the same conditions as were AML cells. As shown in Fig. 3a, no significant effect was detected in cell viability. Thus, bromocriptine treatment spared healthy blood cells. Besides, bromocriptine treatment had no significant effect on the clonogenic capacity of hematopoietic stem/progenitor cells (isolated from lineage-negative umbilical cord blood) both in terms of total number of colonies formed and relative frequency of each colony subtype (Fig. 3b). Taken together, bromocriptine differentially acted as an anti-AML drug that spared the healthy counterpart, especially fighting against the most primitive cell population within the tumor bulk.

## Discussion

The standard treatment of AML has remained essentially unchanged for the last decades, despite advances in molecular biology related to AML and a better understanding of the mechanisms underlying leukemogenesis. De novo drug discovery research involves high development costs, generic competition due to the long time required for reaching clinics that overlaps with legal protection, increasingly conservative regulatory policies, and insufficient breakthrough innovations in the field. Drug repositioning consists in the process of discovering new therapeutic indications for existing approved or candidate drugs, which makes it an attractive drug development strategy, both economically and timely [25]. In order to abrogate the self-renewal capacity of LSCs, a differentiation-inducer already-approved drug was sought using an in silico approach. Bromocriptine, a Parkinsons’ disease, hyperprolactinaemia and galactorrhoea-approved drug, was identified as an anti-AML compound that specifically targets the most primitive fraction of blasts. Interestingly, bromocriptine exerted its cytotoxic effect sparing healthy blood cells.

The original indication for bromocriptine was Parkinsons’ disease with a mechanism of action through dopamine receptors. Bromocriptine acts as a D2 dopamine receptor agonist and a D4 dopamine receptor antagonist [8]. For type II diabetes, a reformulation of bromocriptine was investigated and approved for human use [26]. Although dopamine signaling and metabolism are tightly linked, the exact mechanism of action of bromocriptine in diabetes is partially unknown [27]. More recently, an anti-*Trypanosoma cruzi* effect has been studied for bromocriptine [28], unlikely through dopamine receptor signaling. Taking into account that cancer stem cells (including LSCs) depend on dopamine signaling and its inhibition compromises their viability [29], the anti-AML cytotoxic effect shown here might be exerted by D4 dopamine receptor antagonism and/or other alternative mechanism.

For the last decades, improvements in outcomes for adult AML patients are most likely attributable to advances in supportive care and optimization of hematopoietic progenitor cell transplantation. Relapse continues to be the main reason for AML patients to succumb to the disease. Due to their self-renewal and differentiation capacity, LSCs are thought to be responsible for sustaining and regenerating AML in the patient. Thus, abolishment of the self-renewal capacity is critical for eliminating the disease. Bromocriptine treatment reduced the clonogenic potential of AML cells as determined by CFU assays, the ex vivo “gold standard” test for measuring self-renewal.

In concordance with our results, Liberante and coworkers recently described bromocriptine as a potential drug for myelodysplastic syndromes (MDS) and secondary AML (sAML) [30]. MDS involves different entities that display multilineage dysplasia with ineffective hematopoiesis, developing a sAML in 30 % of patients [31]. Even though sAML constitutes a separated subgroup within AML; sAML and de novo AML share multiple clinical features [17]. However, AML and MDS behave differently from a biological point of view [32]. Indeed, treatment regimens are specific for each disease. 5 out of 11 AML samples analyzed in this study belonged to the AML with myelodysplasia-related changes (AML-MRC). This group includes: (1) AML arising from previous myelodysplastic syndrome (MDS) (sAML) or an MDS/myeloproliferative neoplasm, (2) AML with a specific MDS-related cytogenetic abnormality, and/or (3) AML with multilineage dysplasia [17]. Only 15–40 % of the AML-MRC patients have prior MDS [33–36] and, the clinical outcome of patients with a history of MDS are not significantly different from the remaining cases of AML-MRC [36, 37]. Consequently, the majority of AML-MRC does not evolve from a antecedent MDS. Moreover, the sensitivity to bromocriptine treatment of AML-MRC (bulk population mean survival: 47.33 ± 12.94 %; primitive population mean survival: 24.09 ± 10.72 %) was equivalent to none-AML-MRC samples (bulk population mean survival: 46.21 ± 13.03 %; primitive population mean survival: 23.69 ± 11.63 %). These results suggest that the sensitivity to bromocriptine is not related to the presence of myelodysplasia-related changes although the number of samples analyzed is limited to demonstrate that bromocriptine sensitivity in AML is independent of prior MDS. Accordingly, bromocriptine may target a common signaling pathway shared by myeloid neoplastic cells. Interestingly, this drug differentially decreases clonogenicity and self-renewal capacity of AML cells, a cellular effect difficult to ex vivo evaluate in MDS and critical to definitively eliminate the disease in patients. Hence, our results support its further validation in a clinical setting through a drug repositioning strategy for AML.

## Conclusions

- Bromocriptine is described as a potent anti-leukemia drug that mainly targets leukemia stem cells,
- Bromocriptine treatment spared healthy mature blood cells and hematopoietic stem cells.

## Additional files

10.1186/s12967-016-1007-5 Upregulated probe set upon AML differentiation.
10.1186/s12967-016-1007-5 Downregulated probe set upon AML differentiation.
10.1186/s12967-016-1007-5 Bromocriptine was identified in the in silico screening. A. Bromocriptine molecular structure generated with JMol software. **A** “ball and stick” chemical structure is represented. Balls: atoms (grey, carbon; red, oxygen; blue, nitrogen; purple, bromine); sticks: bonds. **B** CMap results for bromocriptine.
10.1186/s12967-016-1007-5 Bromocriptine treatment induced apoptosis and differentiation in AML cells. **A** MonoMac-1 AML cells were treated with 10 µM Bromocriptine for 48 h. Frequency of Annexin-V positive cells measured by flow cytometry is represented. **B** MonoMac-1 cells were treated with bromocriptine at the concentrations indicated. Frequency of CD4-positive cells detected by flow cytometry is represented. Bars represent mean values of at least 3 experiments performed in triplicates. Error bars represent SEM. ****p<0.001.
10.1186/s12967-016-1007-5 Sensitivity to bromocriptine treatment is prognosis-independent. AML primary patient samples were treated for 72 h with vehicle control or 10 µM bromocriptine. Cell viability was measured by flow cytometry. Primitive population corresponds to the CD34^+^CD38^-^ AML fraction. Live cells refer to control are represented, each symbol type represents an individual AML patient and each symbol corresponds to an independent experimental point. AML samples are classified based on their risk group: green, favorable; yellow, intermediate; red, unfavorable. ***p<0.005; ****p<0.001.

## Abbreviations

AML: Acute myeloid leukemia
LSC: Leukemia stem cells
BM: Bone marrow
MNC: Mononuclear cell
PMA: Phorbol myristate acetate
APL: Acute promyelocytic leukemia
MDS: Myelodysplastic syndrome
sAML: Secondary AML
CFU-B: Colony-forming units-Blasts
