# Supplementary material for: Repositioning of bromocriptine for treatment of acute myeloid leukemia
Source: J Transl Med. 2016 Sep 7;14(1):261. doi: 10.1186/s12967-016-1007-5 (PMC5015257; doi:10.1186/s12967-016-1007-5)
Supplement: Supplementary file 3 — 10.1186/s12967-016-1007-5 Bromocriptine was identified in the in silico screening. A. Bromocriptine molecular structure generated with JMol software. A “ball and stick” chemical structure is represented. Balls: atoms (grey, carbon; red, oxygen; blue, nitrogen; purple, bromine); sticks: bonds. B CMap results for bromocriptine. [file 12967_2016_1007_MOESM3_ESM.pdf]

A

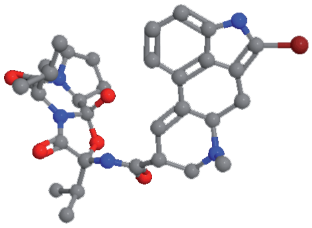

B

Cell line	Origin	Concentration	Score
HL-60	AML	5 μM	0,816
MCF7	Breast cancer	5 μM	0,249
MCF7	Breast cancer	5 μM	0,230
PC3	Prostate cancer	5 μM	0,000
PC3	Prostate cancer	5 μM	-0,482
