# Supplementary figures and images for: Repositioning of bromocriptine for treatment of acute myeloid leukemia
Source: J Transl Med. 2016 Sep 7;14(1):261. doi: 10.1186/s12967-016-1007-5 (PMC5015257; doi:10.1186/s12967-016-1007-5)

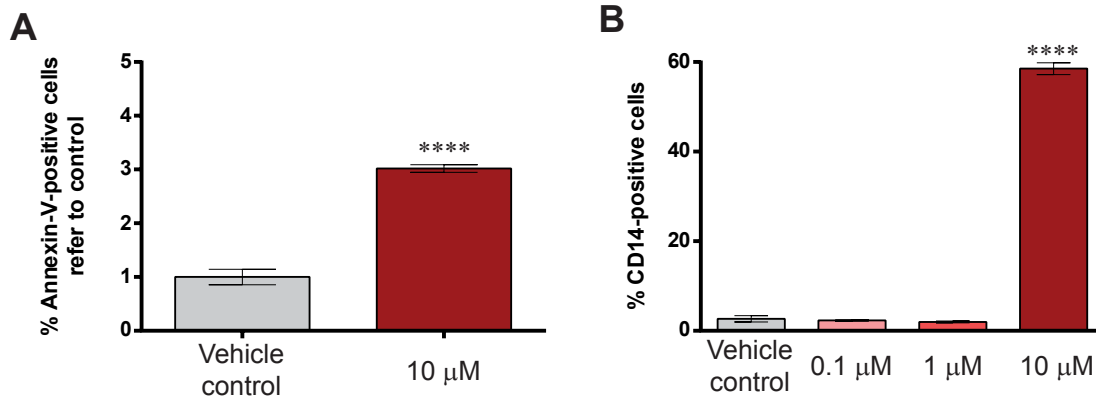

Supplement: Supplementary file 4 — 10.1186/s12967-016-1007-5 Bromocriptine treatment induced apoptosis and differentiation in AML cells. A MonoMac-1 AML cells were treated with 10 µM Bromocriptine for 48 h. Frequency of Annexin-V positive cells measured by flow cytometry is represented. B MonoMac-1 cells were treated with bromocriptine at the concentrations indicated. Frequency of CD4-positive cells detected by flow cytometry is represented. Bars represent mean values of at least 3 experiments performed in triplicates. Error bars represent SEM. ****p<0.001. [file 12967_2016_1007_MOESM4_ESM.pdf]

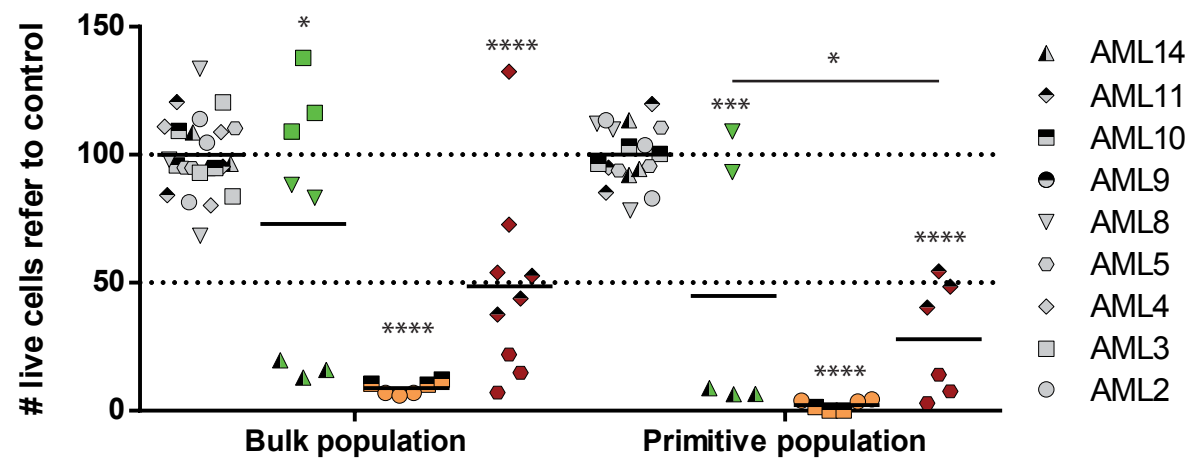

Supplement: Supplementary file 5 — 10.1186/s12967-016-1007-5 Sensitivity to bromocriptine treatment is prognosis-independent. AML primary patient samples were treated for 72 h with vehicle control or 10 µM bromocriptine. Cell viability was measured by flow cytometry. Primitive population corresponds to the CD34+CD38- AML fraction. Live cells refer to control are represented, each symbol type represents an individual AML patient and each symbol corresponds to an independent experimental point. AML samples are classified based on their risk group: green, favorable; yellow, intermediate; red, unfavorable. ***p<0.005; ****p<0.001. [file 12967_2016_1007_MOESM5_ESM.pdf]
